# Supplementary material for: Plasma neurofilament light chain is increased in Niemann-Pick Type C but glial fibrillary acidic protein remains normal
Source: Acta Neuropsychiatr. 2024 Mar 27;37:e20. doi: 10.1017/neu.2024.14 (PMC13130257; doi:10.1017/neu.2024.14)
Supplement: Eratne et al. supplementary material [file S0924270824000140sup001.docx]

# Supplementary Material

**COLLABORATORS:**

**On behalf of others in The MiND Study Group:**

| **Name** | **Affiliations (separated by semi colon)** |
| --- | --- |
| Christa Dang | National Ageing Research Institute, Melbourne, Australia |
| Matthew Kang | Neuropsychiatry, Royal Melbourne Hospital;  Melbourne Neuropsychiatry Centre & Department of Psychiatry, University of Melbourne;  Psychiatry, Alfred Health; |
| Jasleen Grewal | Psychiatry, Alfred Health; |
| Hannah Dobson | Psychiatry, Alfred Health; Neuropsychiatry, Royal Melbourne Hospital |
| Charles Malpas | Melbourne School of Psychological Sciences, University of Melbourne; Department of Medicine, Royal Melbourne Hospital, University of Melbourne |
| Rosie Watson | Population Health and Immunity Division, The Walter and Eliza Hall Institute of Medical Research, Parkville, Australia;  Department of Aged Care and Medicine, The Royal Melbourne Hospital, University of Melbourne, Parkville, Australia |
| Nawaf Yassi | Population Health and Immunity Division, The Walter and Eliza Hall Institute of Medical Research, Parkville, Australia;  Department of Medicine and Neurology, Melbourne Brain Centre at the Royal Melbourne Hospital, University of Melbourne, Parkville, Australia |
| Terence J. O’Brien | Department of Neuroscience, The Central Clinical School, Monash University |
| Patrick Kwan | Department of Neuroscience, The Central Clinical School, Monash University |
| Samuel F Berkovic, MD, FRS | Epilepsy Research Centre, Department of Medicine, Austin Health, The University of Melbourne, Heidelberg |
| Christos Pantelis | Melbourne Neuropsychiatry Centre & Department of Psychiatry, University of Melbourne & Melbourne Health;  Mid West Area Mental Health Service, Melbourne Health |
| Oskar Hansson | Department of Clinical Sciences, Clinical Memory Research Unit, Faculty of Medicine, Lund University, Lund/Malmö, Sweden |
| Shorena Janelidze | Department of Clinical Sciences, Clinical Memory Research Unit, Faculty of Medicine, Lund University, Lund/Malmö, Sweden |
| Qiao-Xin Li | National Dementia Diagnostics Laboratory, The Florey Institute, University of Melbourne |
| Christiane Stehmann | Australian National CJD Registry, The Florey Institute, Melbourne |
| Christopher Fowler | The Florey Institute of Neuroscience and Mental Health, The University of Melbourne, Parkville, VIC |
| Sarah Farrand | Neuropsychiatry, Royal Melbourne Hospital; Melbourne Neuropsychiatry Centre & Department of Psychiatry, University of Melbourne |
| Michael Keem | Neuropsychiatry, Royal Melbourne Hospital |
| Cath Kaylor-Hughes | Integrated Mental Health Team  Dept of General Practice  University of Melbourne, 780 Elizabeth St, Melbourne, 3010 |
| Richard Kanaan | Dept of Psychiatry, University of Melbourne, Austin Health, Heidelberg, VIC 3084 |
| Piero Perucca | Department of Medicine, Austin Health, The University of Melbourne; Comprehensive Epilepsy Program, Austin Health; Department of Neuroscience, Central Clinical School, Monash University; Department of Neurology, The Royal Melbourne Hospital; Department of Neurology, Alfred Health, Melbourne, VIC, Australia |
| Rashida Ali | Alfred Health; Monash University |
| Colin L Masters | National Dementia Diagnostics Laboratory, The Florey Institute, University of Melbourne |
| Steven Collins | Australian National Creutzfeldt-Jakob Disease Registry, Florey Institute of Neuroscience and Mental Health and Department of Medicine, The University of Melbourne. |
| Andrew Evans | Neuropsychiatry, Royal Melbourne Hospital |
| Anna King | The Wicking Dementia Centre, Tasmania |
| Jane Gunn | Department of General Practice, The University of Melbourne |
| Tianxin Pan | Health Economics Unit \| Centre for Health Policy \| Melbourne School of Population and Global Health, The University of Melbourne |
| Ilias Goranitis | Health Economics Unit \| Centre for Health Policy \| Melbourne School of Population and Global Health, The University of Melbourne |
| Steve Simpson-Yap | CORe, The Royal Melbourne Hospital, The University of Melbourne, Melbourne, VIC, Australia |
| Tomas Kalincik | CORe, The Royal Melbourne Hospital, The University of Melbourne, Melbourne, VIC, Australia; Neuroimmunology Centre, Department of Neurology, The Royal Melbourne Hospital, Melbourne, VIC, Australia |
